# Inequality embodied: A systematic review of the spatial and temporal variation of cortical bone health measures and socioeconomic status

**DOI:** 10.1016/j.bonr.2026.101964

**Published:** 2026-10-20

**Authors:** Phoebe Meyrick, Laura A.B. Wilson, Justyna J. Miszkiewicz

**Affiliations:** a School of Social Science, University of Queensland, St Lucia, Brisbane, Australia; b School of Archaeology and Anthropology, The Australian National University, Canberra, Australia; c School of Biological, Earth and Environmental Sciences, UNSW Sydney, Australia; d Earth, Life, Time Group, Naturalis Biodiversity Center, Leiden, the Netherlands

**Keywords:** Inequality, Osteoporosis, Bone remodelling, Evolution, Epidemiology

## Abstract

**Background:**

Like many other chronic conditions, osteoporosis has been proposed to follow a social gradient where socio-economic disadvantage is associated with a higher diagnosis and fracture risk. While evidence for this association has come from biomedical research, and independent observations from studies on ancient human remains, the relationship between measured socioeconomic status (SES) and various parameters of ‘bone health’ appears inconsistent across different populations, methods, and timeframes. In this review article, we apply a comprehensive social and evolutionary framework to assess the spatial and temporal variation of SES on bone health.

**Methods:**

A systematic review of the current biomedical and archaeological literature examined the association between SES and bone health. Peer reviewed articles were obtained through Google Scholar and PubMed. Four quantitative indicators of bone health were considered: bone mineral density (BMD), cortical index, osteoporotic fractures, and histomorphometry.

**Results:**

Forty articles were identified, of which 30 reported a significant association between low SES and low BMD and/or high fracture risk. The earliest evidence for socially patterned osteoporosis was possibly in Ancient Egypt (2687–2191 BCE).

**Conclusion:**

The examined literature generally supported the association between social disadvantage and poor bone health outcomes, following the social gradient. However, we found that research on both modern and archaeological skeletal samples does not always account for intraskeletal variability or use comparable methods when assigning SES, creating between-study inconsistencies. We highlight the contributions of archaeological studies on social conditions and bone fragility, emphasising that socially patterned osteoporosis is not a solely modern-day occurrence.

## Highlights

• Ancient and modern populations show evidence for social gradient of osteoporosis.
• Evidence for socially stratified bone health since at least Ancient Egypt
• Temporal and geographical gaps in research on social patterns of osteoporosis exist.

## 1 Introduction

Osteoporosis is often considered a modern disease based on the extended life expectancy of people today, although cases of increased fragility and bone loss extend back to prehistoric populations (Perzigian, 1973; Zaki et al., 2009). Osteoporotic fractures due to the loss of bone and structural deterioration have become a social and economic burden in recent years (Burge et al., 2007; Hernlund et al., 2013; Shen et al., 2022). A Social Determinants of Health (SDoH) (World Health Organization, 2008, World Health Organization, 2025) lens applied to skeletal conditions recognises the role that socioeconomic inequality plays in health outcomes (Brennan-Olsen et al., 2019). The SDoH framework engages with the idea that lifestyle and structural factors outside of our control influence health through inequal access to essential resources such as education, healthcare, nutrition, housing and occupational opportunities, and predisposes those in disadvantaged socioeconomic positions to poorer bone health outcomes, especially when social marginalisation is experienced during key skeletal formation phases such as peak bone mass accrual in the first three life decades (Brennan-Olsen et al., 2019). A social gradient of osteoporosis has been recognised with lower socioeconomic groups often demonstrating higher rates of fractures when compared to groups of higher status (Brennan et al., 2015; Brennan-Olsen et al., 2016). Despite this gradient, biomedical studies have either continued to report mixed conclusions on the association between bone loss and socioeconomic status (SES) or, as has been identified in previous systematic reviews, have had limited engagement with SES as a factor in understanding ‘bone health’ (Brennan et al., 2015; Brennan-Olsen et al., 2016). Bone ‘health’ is a general term used to describe the various properties of bone tissues, including but not limited to strength, density, and structural integrity (Office of the Surgeon, 2004). Here, we are using this term to encompass both bone quality – strength and resistance as a result of the composition of collagen and bone mineral matrix (Compston, 2006), and quantity – bone mass and/or mineral density as a measure of the amount of bone tissue in the skeleton (Docaj and Carriero, 2024).

In those biomedical studies where social factors have been accounted for when researching osteoporosis, one of the most prevalent sources of between-study heterogeneity and incomparability is the methods used in determining SES. This is not a strictly biomedical problem as sociologists and epidemiologists have discussed issues with measuring SES for decades (Brennan et al., 2009b, Brennan et al., 2011; Valentin et al., 2021). Across biomedical literature, SES has been assigned at multiple levels, including individual, household, suburb, and regional, using a range of lifestyle and systemic/structural non-standardised factors such as education, employment, income, housing, and marital status, which are sometimes independent and can relate to each other in indirect ways (e.g., Beauchesne and Agarwal, 2014; Martin and Armelagos, 1979; Mays et al., 1998). Past reviews in this area have highlighted these inconsistencies, but there is no readily available alternative (e.g., Kneissel et al., 1994; Mays, 1996, Mays, 2000), although sociologists have proposed alternative paths, such as reconceptualisation of SES as a set of socioeconomic conditions rather than compressing it into abstract indices that still require validation in clinical contexts (e.g., Antonoplis, 2023). This issue is also present in examinations of SES and bone health in archaeological populations where SES designations are often undertaken through funerary characteristics, such as burial location or grave goods interpreted to indicate one's status during life.

While earlier studies have attempted to map general bone loss in the past to investigate its origins and patterns, inconsistent results were noted due to issues with bone mineral density (BMD) and fracture analysis in archaeological human remains (Agarwal, 2021; Mays, 1999; Waldron, 2020). These measures can be confounded by post-mortem chemical and structural bone alteration, environmental damage, misapplication of clinical standards, and a lack of methodological standardisation (Brickley and Agarwal, 2003; Kranioti et al., 2019). Studies employing metacarpal cortical index (MCI), femoral cortical index (FCI), fragility fractures, and histomorphometric measures in samples from prehistoric Nubia (350–550 CE), 3rd–4th century England, and Imperial Rome have reported increased bone loss than seen in modern populations (Beauchesne and Agarwal, 2014; Martin and Armelagos, 1979; Mays, 2006). However, MCI and dual-energy X-ray absorptiometry (DXA) at the proximal femur both showed bone loss in a sample from rural medieval England sample to follow age- and sex-related patterns of bone loss in modern populations (Mays, 1996; Mays et al., 1998), and trabecular bone and DXA studies at the lumbar spine showed the same in 4000-year-old skeletal remains from Austria (Kneissel et al., 1994). While femoral DXA studies from 11 to 19th century Poland and 14–15th century Sweden showed higher BMD than modern populations (Bajon et al., 2006; Ekenman et al., 1995). These inconsistencies may arise from applying a predominantly biological framework in BMD analyses, such as examining its variation through an age and sex lens only (see Agarwal (2021) for discussion), and implementing an additional SDoH perspective may provide more information on patterns of BMD variation in the past.

Prior research shone an inter-disciplinary light on the SDoH perspective on bone health by highlighting that SES compromised cortical bone microstructure in medieval populations ruled by feudalism (Miszkiewicz and Cooke, 2019; Miszkiewicz and Mahoney, 2016; Miszkiewicz et al., 2019). This research used static bone histomorphometry in femoral bone from a medieval cemetery showing that historical socioeconomic hierarchies were reflected in physically demanding labour and poor nutrition as cumulative stressors for disadvantaged populations leading to long term issues with bone remodelling. As this research focused on medieval contexts, it raises the question as to whether populations from other time periods with SES structures might have experienced similar bone problems. Since there is yet to be a systematic review of the associations of social status and bone health in both archaeological and modern populations, this paper consolidates this evidence to evaluate the temporal and spatial variations of different bone health measures and their relationship to SES.

## 2 Materials and methods

### 2.1 Eligibility criteria

This systematic review complies with the Preferred Reporting Items for Systematic Review and Meta-Analysis (PRISMA) statement (Page et al., 2021) (Supplemental Table 1), but was not registered in the International Prospective Register of Systematic Reviews (PROSPERO) as our study includes archaeological papers that do not meet all the reporting criteria related to health-related outcomes. The applied eligibility criteria are listed in Table 1.

**Table 1:** Eligibility criteria applied to the search strategy used in this review.

Inclusion criteria:	Exclusion criteria:
• Peer-reviewed, published journal articles with an archaeological or biomedical focus • Any study designs • Measures of cortical bone health: histomorphometry, cortical index, BMD, osteoporotic fracture incidence (described in Table 2). • Studies on adult populations • Populations divided into socioeconomic groups.	• Grey literature (including conference abstracts, preprint articles, theses/dissertations government documents). • Review articles • Articles in languages other than English • Studies on trabecular bone and jaw bone in dental health studies (e.g. alveolar bone). • Studies on adolescents or children.

We only included peer-reviewed journal articles reporting new data, therefore review articles and grey literature resources were excluded. Given some of the analytical issues with BMD and fractures in archaeological samples outlined above, we aimed for our review to scope a wider range of ‘bone health’ measures that include cortical bone histomorphometry and cross-sectional geometry characterising bone tissue distribution. This excluded pure trabecular bone research so that the biomedical and archaeological papers can be meaningfully compared, as trabecular bone structures in archaeological samples can undergo soil infiltration and preservation issues (Brickley and Agarwal, 2003; Mays, 1999). We do, however, acknowledge that BMD scans typically give a reading from combined cortical and trabecular bone regions, which means the BMD measures considered in our review do still partially represent trabecular bone. We also aimed to capture research on adults rather than adolescents or children so that bone modelling that occurs with growth during early ontogeny does not conflate bone quantity and quality that arises predominantly from remodelling in adulthood. Our review also excluded research that assessed jaw bone health in dental studies (e.g. alveolar bone research).

The inclusion criteria were narrowed down to studies that examined quantitative indicators of cortical bone quantity and/or quality (Table 2): cortical index, BMD, osteoporotic fractures, and cortical bone histomorphometry (including measures such as secondary osteon population density (OPD), and geometric properties of secondary osteons, such as size of Haversian canals or area of secondary osteons). These bone parameters were assessed alongside the term ‘socioeconomic status’ or its abbreviation of ‘SES’ (Table 3, including iterations such as ‘disadvantage’, ‘poverty’, and ‘affluent’). For data synthesis the eligible articles were grouped into biomedical and archaeological studies and assessed by cortical bone indicator and exposure (e.g. DXA scan for BMD, fracture incidence for osteoporotic fractures), country and year of examination, SES designation, and study design (see Supplemental Table 4).

**Table 2:** Summary of terms, their definition and justification for our systematic review.

Keywords	Definition and justification for inclusion in systematic review
Cortical index	Unitless value calculated by measuring cortical bone width minus medullary width/total bone width (Mays, 1996). In biomedical studies, cortical index has been previously used to assess bone fragility and predict fractures in the elderly population.
Cortical bone histology/histomorphometry (secondary osteon population density, secondary osteon and Haversian canal geometric properties, osteocyte lacunar densities)	Number of secondary osteons or osteocyte lacunae per square millimetre quantified from 2D ground histology samples (Gocha and Agnew, 2016). Dimensions of secondary osteons and Haversian canals (e.g., area, perimeter) are measured directly from 2D images of cortical bone histology (Miszkiewicz et al., 2026). These provide insights into bone remodelling dynamics and have been previously associated with SES in archaeological populations.
BMD	Reported in g/cm^2^ from DXA scans, commonly at the femoral neck, lumbar vertebrae, and radius. Direct measure of bone mineral content allowing to evaluate bone strength and loss. BMD values have been previously associated with SES in biomedical studies.
Osteoporotic fractures	Bone fractures due to increased fragility, which are commonly used in biomedical research as an indicator of bone health issues and have been previously examined in relation to SES. Prevalence has also been estimated through macroscopic observation at individual and population level in past populations. For population level studies, incidence rates are reported in epidemiological studies (Waldron, 2020).
SES	Measure of an individual and group's standing on a social and economic scale within the broader population, estimated from indicators of education, occupation, income. SES is the key measure used in the social gradient of osteoporosis studies.

**Table 3:** The searched terms and results returned for the systematic review investigating various bone health measures (see Table 1) and socio-economic status (SES).

Search terms	“Cortical index” + SES	“bone mineral density” OR BMD + SES	“osteon population density” OR OPD + SES; Microstructure + SES	Fragility OR osteoporotic AND fracture + SES
Results from PubMed	0	32	17; 1	14
Results from Google Scholar	123	19,700	85; 429	4290

### 2.2 Search strategy

Keyword searches were undertaken separately for each of the bone health indicators and SES combination in PubMed and Google Scholar (Jin et al., 2024; Winn et al., 2026) covering studies published until April 2026. The reference lists of all the studies that matched the inclusion criteria were later also searched for any additional articles that did not appear in the original database searches

### 2.3 Quality assessment and analysis

As is standard for systematic reviews, each of the studies was subject to a quality assessment that aimed to measure the bias and validity of the findings within the relevant field (Shaheen et al., 2023). As we included both biomedical and archaeological papers, they underwent separate quality assessments due to their inherent differences in study design, use of statistics and relevant bias influences, such as the use of sex and age estimation methods to obtain demographic information from skeletal remains in archaeology, versus having documented age and sex for patients or cohorts in biomedicine. For the articles we reviewed that were categorised as biomedical, we applied modified quality assessment criteria from Brennan et al. (2009b), Brennan et al. (2011), and Dufault and Klar (2011) (Supplemental Table 2). These articles were divided by study design and subject to criteria on study population, risk factors, outcomes, and analysis and data presentation – gaining one point for each criterion. The resulting scores ranged from out of 10 for cross-sectional studies, out of 12 for cohorts, 13 for case-controls, and 15 for ecological cohorts. For all other articles that were of archaeological nature, we adapted the quality assessment items created by Rajbhoj et al. (2022) specifically for investigating archaeological human remains (Supplemental Table 3). This assessment held sixteen criteria, examining aims and outcomes, sample preservation, methods, statistical tests, and comparative reporting. Each criterion was worth one point, articles achieving half a point if the requirement was reported but inadequate. Therefore, archaeological studies were scored out of 16. Following the quality assessment, we quantified how many articles found positive associations between bone health measures and SES and mapped the studies to generate a temporal and spatial overview of the studies.

## 3 Results

Through the key word searches we initially obtained over 24,000 results from Google Scholar and PubMed combined. Following title/abstract screening, 40 studies fit the inclusion criteria, 35 biomedical and five bioarchaeological (Fig. 1, Table 3). All of the identified studies are cited in the text throughout the results and discussion, however, a complete and compiled list of the references to these studies including their characteristics, study design and bias assessment, can be found in Supplemental Table 4.

**Fig. 1:**
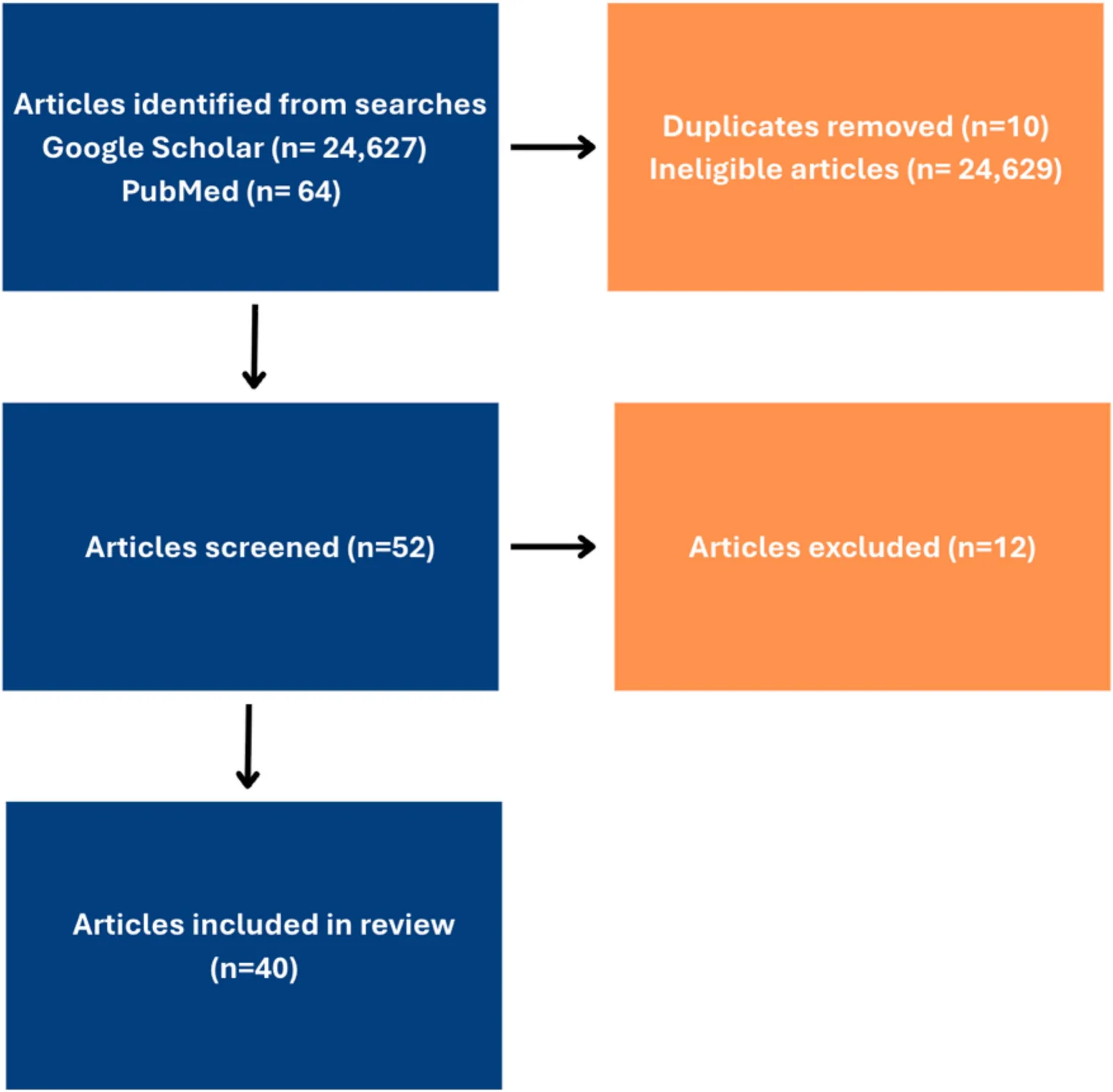
Flow diagram summarising systematic review search results and screening (also see Table 3 for more detailed results).

Only one study (Miszkiewicz and Mahoney, 2016) investigated bone microstructural measures as an indicator of bone health in relation to SES, two (Borrè et al., 2015; Johnson et al., 2019) used cortical index measurements combined with another indicator (hip BMD and radius fractures respectively), while 19 examined DXA-derived BMD alone (Alver et al., 2007; Amiri et al., 2008; Brennan et al., 2010, Brennan et al., 2009a; Crandall et al., 2012; del Rio Barquero et al., 1992; Du et al., 2017; Elliot et al., 1996; Ersoy et al., 2018; Myong et al., 2012; Tajik et al., 2014; Tsai, 2019; Wang and Dixon, 2006; Xiao et al., 2022b; Yasein et al., 2016; Zhang et al., 2023), nine assessed fracture incidences (Brennan et al., 2015), and nine considered both BMD and fractures (Amin et al., 2026; Brennan et al., 2013; García-Quintana et al., 2024; Mahmud et al., 2024; Navarro et al., 2009, Navarro et al., 2013; Park et al., 2016; Pearson et al., 2004; Syddall et al., 2012). Each of the biomedical studies used a different combination of SES indicators to assess their cohort: SES was measured by education level (13 studies), income (11), marital status (5), occupation (7), housing type (3), two were self-reported, one used car availability, and one used medical beneficiary and medical aid records. Lifestyle and health variables (such as smoking and alcohol intake, and physical activity and body mass respectively) were included in 68.5% of biomedical studies (24/35), therefore 11 did not account for any lifestyle or behavioural factors (Brennan et al., 2015; del Rio Barquero et al., 1992; Guilley et al., 2011; Icks et al., 2009; Johnson et al., 2019; Myong et al., 2012; Park et al., 2016; Petit et al., 2017; Quah et al., 2011; Tsai, 2019; Zingmond et al., 2006). Twenty-one papers used poverty indices (most often in conjunction with other indicators) that calculated SES based on residential address or census area data. In contrast, all five of the archaeological studies used burial location as the status indicator for their assemblages (Borrè et al., 2015; Brødholt et al., 2023, Brødholt et al., 2022; Miszkiewicz and Mahoney, 2016), and one also used grave goods in combination with location (Zaki et al., 2009). Six (Elliot et al., 1996; García-Quintana et al., 2024; Navarro et al., 2009, Navarro et al., 2013; Park et al., 2016; Zingmond et al., 2006) biomedical studies used one indicator to determine SES, meaning 83% of the biomedical studies accounted for multiple factors (counting poverty indices as measuring more than one).

The biomedical studies consisted of 24 cross-sectional studies, two case-cohort studies, six cohort studies, and three ecological cohort studies. The bias and consequent quality of the biomedical studies ranged from 50% to 92%, with a mean of 64% and median score of 61.5% (Supplemental Table 4). The bioarchaeological studies scored between 84% and 94%, with a mean of 88.8% (Supplemental Table 4).

Over half of the studies (25/40) revealed positive linear associations between lower BMD, higher fracture risk and lower SES in disadvantaged groups, concluding that higher education, income, and health literacy are factors that influence better outcomes for bone health (Supplemental Table 4). A further five studies reported significant positive associations between low BMD/higher fracture risk and SES, but only in females (Alver et al., 2007; Brennan et al., 2013; Brødholt et al., 2022; Guilley et al., 2011; Zaki et al., 2009). Of the studies that resulted in negative associations—where people in higher socioeconomic positions had lower BMD or higher fracture risk—or where there were absent associations, some biases in methods or samples examined caused unexpected results. The two studies (Borrè et al., 2015; Johnson et al., 2019) that employed cortical index measures had no significant results using that method but found associations with SES between their other measures of bone health, femoral neck BMD and distal radius fracture incidence.

### 3.1 Temporal and spatial variation

The biomedical studies examined cohorts between 1939 and 2025, with 68.5% of the studies extracting data from post 2000. Whereas the archaeological studies examined skeletal remains representing individuals from various time periods, spanning approximately 2687 BCE (4713 years ago) to 1567 CE (459 years ago), predictably focusing on populations from the medieval period (980 to 1567 CE) ruled by feudalism. Together these studies leave a gap of around 1500 years between the end of the Old Kingdom and the beginning of the medieval period, and ~ 370 years between the late sixteenth and mid-twentieth centuries. There are few studies of populations from the southern hemisphere, with marked gaps in South America, Africa and Oceania (Fig. 2).

**Fig. 2:**
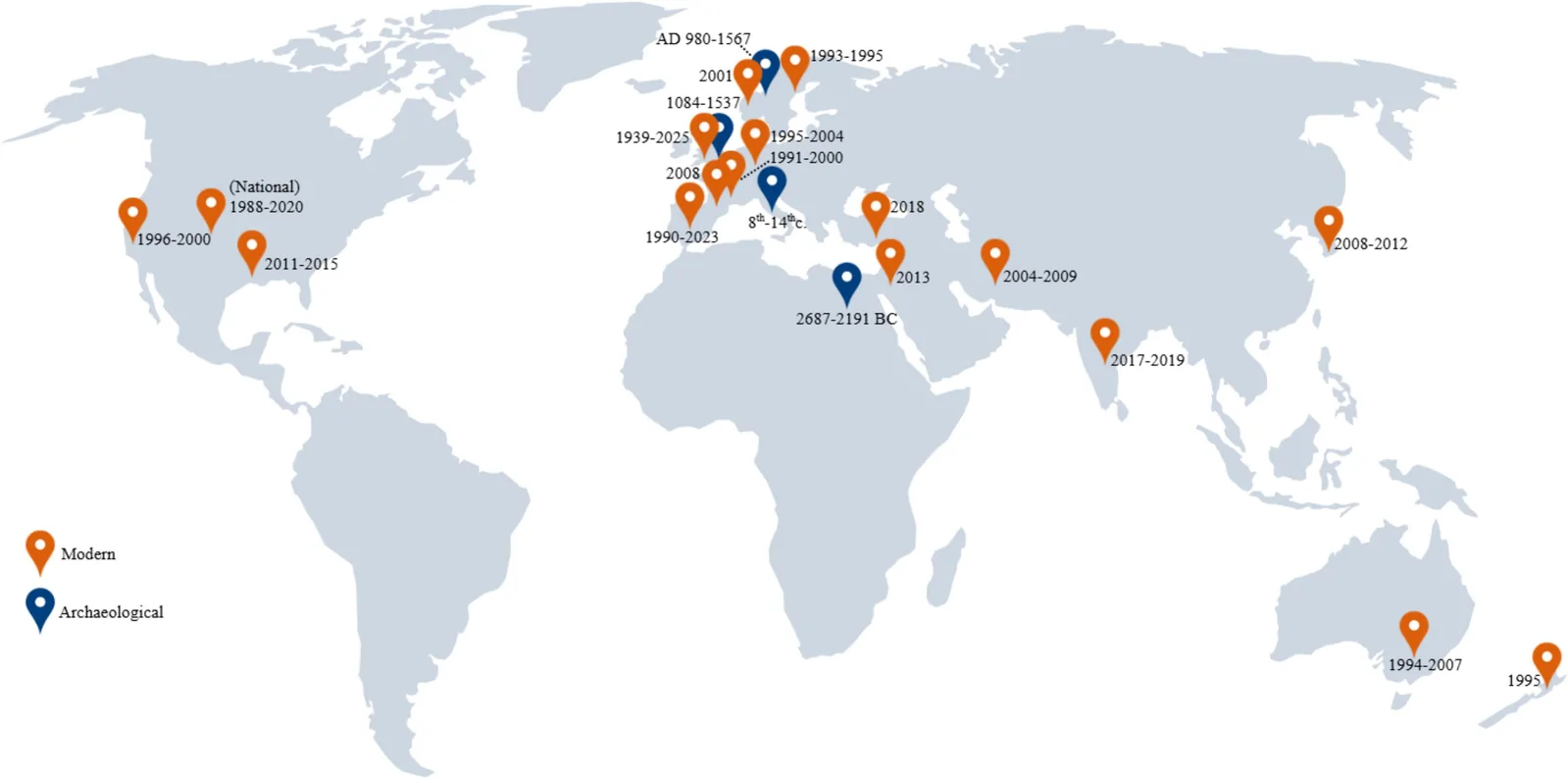
Map marked with locations of the countries and time period examined in studies of bone health and socio-economic status, bioarchaeological studies in blue and biomedical studies in orange. Only one marker was placed for each country even if multiple studies were undertaken there.

## 4 Discussion

The biomedical and archaeological papers reviewed here generally indicate, with some exceptions, that there is a significant association between various measures of poor bone health (e.g., low BMD, high fracture risk) and a disadvantaged socioeconomic position inferred from SES. Although not all these studies examined osteoporosis diagnosis outcomes specifically, the overall investigation of bone quality and quantity characteristics increasing osteoporosis risk align with the social gradient for osteoporosis framework predictions. However, aside from the conceptual findings, evaluating these studies has highlighted methodological and interpretive issues that seem to impact both biomedical and archaeological contexts, such as sample representativeness, not accounting for intraskeletal variability, and between study variation in SES designation based on limited or varied social and economic variables/data. Despite these limitations, our review stresses that contemporary and archaeological perspectives on a condition commonly thought to be a modern-day disease reveals that the connectedness of social conditions and bone health has historical roots.

### 4.1 Historical evidence for the social patterning of osteoporosis

Osteoporosis is often thought to be an issue of the modern world, rooted in our broadly sedentary lifestyle conditions, extended life expectancy, and some argue our higher evolutionary risk i.e. adaptations to digest milk, loss of skin pigment outside Africa for vitamin D absorption (Bortz, 1989; Grob, 2014; Karasik, 2008; Walsh, 2015). The medical diagnosis of osteoporosis as we understand it today is relatively new, with diagnostic standards and agreed definitions not developed until 1994 and 2001 respectively (Lorentzon and Cummings, 2015; National Institutes of Health, 2001; World Health Organization, 1994), leading to an increase in diagnoses and the recognition of osteoporosis as a major medical problem (Grob, 2014). This ‘recent’ understanding of the condition may contribute to its labelling as a modern phenomenon, although previous archaeological reviews have highlighted that osteoporosis was present in past populations (Agarwal, 2021; Mays, 1999; Tabor et al., 2023). Therefore, in addition to evolutionary changes, longevity, lifestyle and other factors (Karasik, 2008; Madimenos, 2015), the SDoH approach and archaeological perspectives have identified social and economic patterning of bone health quantity and quality present since at least 5000 years ago in Old Kingdom Egypt, and prominence in the medieval period (Agarwal, 2021; Mays, 1999; Tabor et al., 2023; Zaki et al., 2009). Intact skeletal remains from this long ago are rare, especially when considering preservation rates and applying the necessary exclusionary criteria for absorptiometry (Brickley and Agarwal, 2003; Mays, 1999), making this a significant point for our understanding of bone health outcomes related to social standing in humans.

### 4.2 A life course approach to osteoporosis

Studies that examine both indicators of bone health and SES are currently lacking a longitudinal approach, although this is a recommended practice (Hanson et al., 2018; Harvey et al., 2014). Alongside the SDoH, the Developmental Origins of Health and Disease (DOHaD) and life course approaches are essential epidemiological frameworks for unpacking the long-term effects of unequal social conditions on skeletal health, as they both focus on how poor environments (whether that be intrauterine or postnatal) during development, even for short periods, can become biologically embedded and have long-term consequences (Agarwal, 2016; Barker, 2007; Gowland, 2015). While a DOHaD perspective has been applied to osteoporosis in both biomedical and archaeological contexts, highlighting how adverse early life conditions could increase risk of lower BMD and osteoporotic fractures (Backström et al., 2005; Bowers et al., 2024; Riancho and Brennan-Olsen, 2017; Shere et al., 2019; Wood et al., 2015; Zheng et al., 2018), an interdisciplinary life course approach integrating SES and indicators of cortical bone health is yet to be undertaken. We single out cortical bone here as it facilitates the examination of secondary osteons as products of Basic Multicellular Units and thus allows to capture potential variation in compact bone remodelling with SES (Miszkiewicz and Mahoney, 2016; Miszkiewicz et al., 2026). In archaeological contexts, we are aware of one study (not identified in our review), where higher frequencies of dental markers of childhood developmental stress (linear enamel hypoplasia, LEH) showed preliminarily statistical associations with higher OPD data in high SES medieval English individuals, potentially hinting that early life illness experiences are compensated for through higher cortical bone density in later adulthood (Walker et al., 2019). This implies that high SES might provide a health buffer in line with the social gradient framework. However, such archaeological studies probably should not be labelled as life-course approaches in an epidemiological sense since the individuals are not truly studied over the course of their lifespan. There is also the issue of using LEH alone as a proxy for experiences of illness, since it is a non-specific marker indicating more than assumed experiences of poor health (Towle and Irish, 2020). Nevertheless, since social status was potentially more rigid in the past than today, especially under the feudal law, exemplified by studies of medieval bone health and society (Borrè et al., 2015; Brødholt et al., 2023, Brødholt et al., 2022; Miszkiewicz and Mahoney, 2016), archaeological populations may provide a springboard for examining socioeconomic influences across the life course on the pathogenesis of osteoporosis in present day.

### 4.3 Spatial and temporal variation in investigating bone health and socio-economic status

Our review has identified spatial and temporal gaps in our understanding of the association between bone health and SES. There are two clear gaps between 2191 BCE and 980 CE, and between 1567 and 1939 (Fig. 2). These missing periods encompass influential socioeconomic change in human history, with the latter including some of the most important and polarising changes, colonialisation and industrialisation. Previous bioarchaeological studies have shown that industrialism had major health impacts, embodied in the skeleton through increased childhood stress and mortality (Lewis, 2002, Lewis, 2013; Newman and Gowland, 2017). Major economic change and inequality was also driven by colonialism redistributing power relationships, starting from 1402 and culminating in the 19th century, with health implications lasting until the present day (Aldrich and Johnson, 2018; Robinson and Acemoglu, 2017; Yuni et al., 2025). Gathering analyses on archaeological populations from these eras, especially from between the medieval and recent historical periods, may provide vital information on how socioeconomic change and inequality is embodied in the skeleton. Additionally, there are only five bioarchaeological papers on SES and various measures of bone health, with four of them from Europe. This geographical pattern is also reflected in the biomedical studies as they are mostly grouped in North America and Europe, with no studies identified in our review from South America, Africa, Southeast Asia or the Pacific. Historically, the nature of studies performed have skewed the epidemiology of osteoporosis and perspective of risk towards North American and European populations (Grob, 2014), but recent investigations have shown that the distribution of risk is more varied and possibly even more prevalent in developing countries (Handa et al., 2008; Kruger and Wolber, 2016; Xiao et al., 2022a). While the repository of osteoporosis studies in some of these regions is limited but growing (Chandran et al., 2023; Liu et al., 2025; Mohd-Tahir and Li, 2017), there remains a paucity of primary studies and census data from Africa and South America (Paruk et al., 2021; Ward et al., 2023). These papers call this sparsity to attention and urge for improved access to care and greater awareness at the institutional level, for example, by including osteoporosis in medical school curriculum and having wider access to diagnostic equipment (Paruk et al., 2021; Ward et al., 2023). While a historical focus on North America and Europe may explain lack of data for other regions, there remains a need for increased osteoporosis awareness and healthcare infrastructure in these populations from which to build a foundation of care and implement an SDoH approach.

Across the geographical distribution of studies that we have identified, modern data are more widespread than archaeological data and have shown influences in lifestyle that alter bone health aside from SES. For instance, diet and nutrient availability varies with culture, this has been exemplified in studies of Iranian women wearing head coverings having less vitamin D than other groups and experiencing lower BMD not solely as an effect of SES (Tajik et al., 2014; Amiri et al., 2008); or farmers being exposed to agricultural pesticides that negatively impact BMD as a consequence of their occupation rather than wealth (Singh et al., 2021). This emphasises the importance of accounting for cultural variations as well as economic status to fully understand the interaction between biology and society in the skeleton. In bioarchaeological studies, this variation may be harder to grasp and can be controlled for by assessing socioeconomic groups that would have likely experienced the same environment, proximity to resources, and infection regardless of status i.e., examine groups from within the same community, burial location, or region as opposed to across countries or geographical barriers. The SDoH approach provides a framework that acknowledges that chronic stressors and lifestyle factors affect different individuals and groups differently, and also impact these individuals' ability to respond to these stressors. In bone health, there is an interaction between social and biological factors, the nature of which is context specific, which is relevant in both modern and archaeological populations.

### 4.4 Limitations and biases in the current literature

We have identified several areas of bias that are inherent in the current studies of the association between bone health and SES that could be contributing to inconsistencies between studies and negative associations. The main complication is not accounting for intraskeletal variability. Where bone health measures are typically captured from skeletal region or bone, they do not account for the remainder of the skeleton, and that is problematic as different bones have variable remodelling rates e.g. peripheral areas remodel at a higher rate than larger long bones such as the femur (Parfitt, 2021; Stewart et al., 2015). The bone region selected for measurement has a considerable impact on the study outcomes since there will be intra-skeletal variation in remodelling even with one individual. Both biomedical and bioarchaeological studies have shown more significant associations between SES and BMD when assessing BMD at the lumbar spine compared with other locations, namely the proximal femur (Borrè et al., 2015; Crandall et al., 2012; Navarro et al., 2009; Wang and Dixon, 2006; Zaki et al., 2009). Crandall et al. (2012) and Borrè et al. (2015) discuss this difference and posit that it may be due to the ratio of cortical to trabecular bone at the two anatomical sites, the proximal femur having a higher amount of cortical bone, subject to weight bearing and susceptible to physical activity, whereas the lumbar spine is mostly composed of trabecular bone and more sensitive to metabolic and hormonal changes. This may suggest that data on BMD from the femoral neck alone are insufficient and should be supplemented with other skeletal regions, and the lumbar vertebrae may be a reliable addition. However, this may limit studies on archaeological human remains since they are often predisposed to examining the femur given its robusticity and preservation compared to the vertebrae, and add additional sampling burden for modern studies as diagnostic standards are based on the BMD at the femoral neck.

Issues with sample representativeness also permeates both the biomedical and archaeological disciplines, and is especially pertinent when establishing differences between different social groups. Biomedical studies are shown to be liable to overrepresenting more affluent groups, especially when recruiting participants via their general practictioner or hospital databases which introduces bias towards those with access to healthcare (e.g., del Rio Barquero et al., 1992; Guilley et al., 2011; Pearson et al., 2004; Syddall et al., 2012). In archaeological studies that examine social status, the proximity of each of the representative groups must be taken into account, comparing assemblages from across countries or regions possibly introducing fluctuations in diet and physical labour within social groups (Brødholt et al., 2023, Brødholt et al., 2022; Zaki et al., 2009). Bioarchaeologists must also recognise that archaeological human remains only represent a highly selected sample of a wider population (Wood et al., 1992) and consider how burial practices and preservation biases may influence this sample. For example, segregated burials and grave goods commonly, but do not always, differ by social status and relevant historical investigation is necessary, as misidentification of social groups can bias outcomes (Creighton Avery, 2015).

One other aspect of bone health and SES analyses that should be noted in terms of potentially explaining any inconsistencies in outcomes is between study variation in how SES is determined and assigned. The biomedical studies in this review often rely on self-reported and/or area-level census data, although there is a multitude of ways to define SES, for example, using poverty indices, income and unemployment rates, education level, marital status, and residential area (e.g., Brennan et al., 2009a; Elliot et al., 1996; Farahmand et al., 2000; Petit et al., 2017). To have the most accurate representation of SES, most studies either used a predetermined index or combine several factors. Although, studies have used single factors as a proxy for SES, such as education level, and found significant associations between low SES and low BMD/high fracture risk level (Gur et al., 2004; Ho et al., 2005; Holvik et al., 2024; Varenna et al., 1999). SES variables can also differ by individual, family, area level distinctions, and number of SES divisions, with a difference observable between those who used a binary versus more stratified divisions. Two recent BMD studies from the United Kingdom used existing deprivation indices that measure house crowding and unemployment rates, average car and home ownership, income, education, and living environments for a post-code area, and designated patients into either lower or higher SES groups (Amin et al., 2026; Mahmud et al., 2024). Both found positive associations between SES and BMD, and higher fracture risk for more deprived areas, however, by segregating SES into only two groups they potentially obscure nuances in the spectrum of social groups potentially present. In contrast, Brennan et al. (2010) and Brennan et al. (2009a) also examined SES from residential-based poverty indices using very similar factors, yet, they divided SES into quintiles. Due to this, their results showed the highest BMD was in the middle SES quintile. This interesting relationship was attributed to sedentary behaviours in both the unemployed of the lowest SES group, and retirement or easy living of the highest SES group. Should these studies have been evaluated using a binary distribution of SES groups, this pattern may not have been identified. The review undertaken here is also limited by how SES is determined and assigned, as we did not subdivide our analysis by different measures of SES designation due to the high variability of methods used and sample size of our identified papers.

Comparing the influence of SES across time is complicated by the fact that indicators of status differ between archaeological and modern groups. In archaeological papers, SES is often determined by one of a range of status indicators, most often grave goods, burial location and/or burial positioning. In the five papers we identified, all relied on the burial location and its association with a symbol of either high or low status. For example, burial in a church implied a higher degree of wealth, while pauper burials were located separately outside; these papers were supplemented by contextual and historical information such as feudalism in the Middle Ages (Borrè et al., 2015; Brødholt et al., 2023, Brødholt et al., 2022; Miszkiewicz and Mahoney, 2016). In contrast, as demonstrated in Section 3.1, modern forms of SES are designated by factors such as income, marital status, education level, and occupation, which often cannot be determined for archaeological individuals. Additionally, 83% of the identified biomedical papers used more than one indicator to determine SES in their studies, whereas, due to the nature of assigning social groups in the past, four of five of the archaeological studies only used burial location as their indicator of status. This adds a further difficulty in making direct comparisons between modern biomedical and archaeological samples, but forms a foundation from which to explore patterns in bone quantity and quality with SES across time.

There are several areas that future studies could examine to fill in the spatial and temporal gaps identified in this review. The overall quality of the papers, especially from the biomedical field, was average (64%), likely due to the widespread use of retrospective studies and non-blinded designs with low participation from large cohorts. A focus on prospective study designs moving forward could correct some of this inherent bias. When comparing archaeological and biomedical research, the biomedical literature focuses on BMD and osteoporotic fractures as measures of bone health, while cortical index and cortical bone histomorphometry are used minimally. This is likely due to the diagnostic standards used in clinical settings and the limited use of autopsy specimens to investigate this topic, however, there is clearly an opportunity for histomorphometry and cortical index methods to be employed in further bioarchaeological and in biomedical contexts (Miszkiewicz et al., 2026). There is a paucity of bioarchaeological papers in general that examine both SES and indicators of bone loss, which represents an avenue for future studies, any archaeological burial sites with evidence for social stratification can investigate bone health through a socioeconomic perspective. To fill in the temporal gap identified here, we would recommend a life course approach to identify embedded responses to social inequality, studies on archaeological populations from outside Europe, and from between the late 16th and early 20th centuries that may capture the skeletal response to the development of industrial society and wide economic change. Spatial variation in modern populations also requires further study, with extension to populations in the southern hemisphere and historically marginalised groups from these regions – South America, Africa, the Pacific, and Southeast Asia.

## 5 Conclusion

Consolidating both archaeological and modern perspectives of bone health and SES has shown that social patterning may stretch back to Old Kingdom Egypt – highlighting that disadvantaged groups show impaired bone health throughout human history. This confirms that osteoporosis is not a modern-day issue and that there may be a social gradient. The spatial variation in the current literature investigating the association between bone health and SES showed large gaps appearing across most of the Southern Hemisphere, notably in what can be considered developing countries. This variation is a symptom of a larger issue: a lack of primary osteoporosis studies and health structures that can support diagnosis and awareness in these regions, especially Africa and South America. The lack of institutional structures and census data that could provide the template for retrospective study designs, which make up the majority of bone health studies in this review, limit an SDoH approach at this time. However, more studies using an archaeological perspective, especially employing a life course approach and between 1600 and 1900 CE, present the opportunity for deeper understanding of how poor social conditions become embodied as impaired bone quality. Future studies applying standard study designs should be aware of intraskeletal variability and representative biases that may impact the relationship uncovered between SES and bone health. This, and applying underused methods, such as cortical index and histomorphology, may expand our understanding of how socioeconomic inequalities affect the skeleton.

## CRediT authorship contribution statement

**Phoebe Meyrick:** Writing – original draft, Investigation, Formal analysis, Conceptualization. **Laura A.B. Wilson:** Writing – review & editing, Validation. **Justyna J. Miszkiewicz:** Writing – review & editing, Supervision, Methodology, Conceptualization.

## Funding

This work was supported by a University of Queensland Graduate Research School Scholarship; and the 10.13039/501100000923Australian Research Council [FT240100030].

## Declaration of competing interest

The authors declare that they have no known competing financial interests or personal relationships that could have appeared to influence the work reported in this paper.

## Data Availability

No data was used for the research described in the article.
