# Supplementary material for: Inequality embodied: A systematic review of the spatial and temporal variation of cortical bone health measures and socioeconomic status
Source: Bone Rep. 2026 Oct 20;31:101964. doi: 10.1016/j.bonr.2026.101964 (PMC13717043; doi:10.1016/j.bonr.2026.101964)
Supplement: Supplementary file 1 [file mmc1.docx]

Table 1 PRSIMA checklist for the review of bone health and socioeconomic status.

| **Section and Topic** | **Item #** | **Checklist item** | **Location where item is reported** |
| --- | --- | --- | --- |
| **TITLE** | | |  |
| Title | 1 | Identify the report as a systematic review. | ☒ |
| **ABSTRACT** | | |  |
| Abstract | 2 | See the PRISMA 2020 for Abstracts checklist. | ☒ |
| **INTRODUCTION** | | |  |
| Rationale | 3 | Describe the rationale for the review in the context of existing knowledge. | ☒ |
| Objectives | 4 | Provide an explicit statement of the objective(s) or question(s) the review addresses. | ☒ |
| **METHODS** | | |  |
| Eligibility criteria | 5 | Specify the inclusion and exclusion criteria for the review and how studies were grouped for the syntheses. | ☒ |
| Information sources | 6 | Specify all databases, registers, websites, organisations, reference lists and other sources searched or consulted to identify studies. Specify the date when each source was last searched or consulted. | ☒ |
| Search strategy | 7 | Present the full search strategies for all databases, registers and websites, including any filters and limits used. | ☒ |
| Selection process | 8 | Specify the methods used to decide whether a study met the inclusion criteria of the review, including how many reviewers screened each record and each report retrieved, whether they worked independently, and if applicable, details of automation tools used in the process. | ☒ |
| Data collection process | 9 | Specify the methods used to collect data from reports, including how many reviewers collected data from each report, whether they worked independently, any processes for obtaining or confirming data from study investigators, and if applicable, details of automation tools used in the process. |  |
| Data items | 10a | List and define all outcomes for which data were sought. Specify whether all results that were compatible with each outcome domain in each study were sought (e.g. for all measures, time points, analyses), and if not, the methods used to decide which results to collect. | ☒ |
|  | 10b | List and define all other variables for which data were sought (e.g. participant and intervention characteristics, funding sources). Describe any assumptions made about any missing or unclear information. | ☒ |
| Study risk of bias assessment | 11 | Specify the methods used to assess risk of bias in the included studies, including details of the tool(s) used, how many reviewers assessed each study and whether they worked independently, and if applicable, details of automation tools used in the process. | ☒ |
| Effect measures | 12 | Specify for each outcome the effect measure(s) (e.g. risk ratio, mean difference) used in the synthesis or presentation of results. |  |
| Synthesis methods | 13a | Describe the processes used to decide which studies were eligible for each synthesis (e.g. tabulating the study intervention characteristics and comparing against the planned groups for each synthesis (item #5)). | ☒ |
|  | 13b | Describe any methods required to prepare the data for presentation or synthesis, such as handling of missing summary statistics, or data conversions. | ☒ |
|  | 13c | Describe any methods used to tabulate or visually display results of individual studies and syntheses. | ☒ |
|  | 13d | Describe any methods used to synthesize results and provide a rationale for the choice(s). If meta-analysis was performed, describe the model(s), method(s) to identify the presence and extent of statistical heterogeneity, and software package(s) used. | ☒ |
|  | 13e | Describe any methods used to explore possible causes of heterogeneity among study results (e.g. subgroup analysis, meta-regression). | ☒ |
|  | 13f | Describe any sensitivity analyses conducted to assess robustness of the synthesized results. | ☒ |
| Reporting bias assessment | 14 | Describe any methods used to assess risk of bias due to missing results in a synthesis (arising from reporting biases). | ☒ |
| Certainty assessment | 15 | Describe any methods used to assess certainty (or confidence) in the body of evidence for an outcome. | ☒ |
| **RESULTS** | | |  |
| Study selection | 16a | Describe the results of the search and selection process, from the number of records identified in the search to the number of studies included in the review, ideally using a flow diagram. | ☒ |
|  | 16b | Cite studies that might appear to meet the inclusion criteria, but which were excluded, and explain why they were excluded. |  |
| Study characteristics | 17 | Cite each included study and present its characteristics. | ☒ |
| Risk of bias in studies | 18 | Present assessments of risk of bias for each included study. | ☒ |
| Results of individual studies | 19 | For all outcomes, present, for each study: (a) summary statistics for each group (where appropriate) and (b) an effect estimate and its precision (e.g. confidence/credible interval), ideally using structured tables or plots. | ☒ |
| Results of syntheses | 20a | For each synthesis, briefly summarise the characteristics and risk of bias among contributing studies. | ☒ |
|  | 20b | Present results of all statistical syntheses conducted. If meta-analysis was done, present for each the summary estimate and its precision (e.g. confidence/credible interval) and measures of statistical heterogeneity. If comparing groups, describe the direction of the effect. | ☒ |
|  | 20c | Present results of all investigations of possible causes of heterogeneity among study results. | ☒ |
|  | 20d | Present results of all sensitivity analyses conducted to assess the robustness of the synthesized results. | ☒ |
| Reporting biases | 21 | Present assessments of risk of bias due to missing results (arising from reporting biases) for each synthesis assessed. | ☒ |
| Certainty of evidence | 22 | Present assessments of certainty (or confidence) in the body of evidence for each outcome assessed. | ☒ |
| **DISCUSSION** | | |  |
| Discussion | 23a | Provide a general interpretation of the results in the context of other evidence. | ☒ |
|  | 23b | Discuss any limitations of the evidence included in the review. | ☒ |
|  | 23c | Discuss any limitations of the review processes used. | ☒ |
|  | 23d | Discuss implications of the results for practice, policy, and future research. | ☒ |
| **OTHER INFORMATION** | | |  |
| Registration and protocol | 24a | Provide registration information for the review, including register name and registration number, or state that the review was not registered. | ☒ |
|  | 24b | Indicate where the review protocol can be accessed, or state that a protocol was not prepared. | ☒ |
|  | 24c | Describe and explain any amendments to information provided at registration or in the protocol. | ☒ |
| Support | 25 | Describe sources of financial or non-financial support for the review, and the role of the funders or sponsors in the review. | ☒ |
| Competing interests | 26 | Declare any competing interests of review authors. | ☒ |
| Availability of data, code and other materials | 27 | Report which of the following are publicly available and where they can be found: template data collection forms; data extracted from included studies; data used for all analyses; analytic code; any other materials used in the review. | ☒ |

*From:* Page M.J., McKenzie J.E., Bossuyt P. M., Boutron I., Hoffmann T.C., Mulrow C.D., et al. The PRISMA 2020 statement: an updated guideline for reporting systematic reviews. BMJ 2021(372), n71. <https://doi.org/10.1136/bmj.n71> This work is licensed under CC BY 4.0. To view a copy of this license, visit <https://creativecommons.org/licenses/by/4.0/>

Table 2 Quality assessment criterion for clinical studies that investigate the association between bone health and socioeconomic status.

| **Item** | **Criterion** | **C/CC/CS/EC*** |
| --- | --- | --- |
| Study population | | |
| 1 | Selection before disease was present or at uniform point | C/CC/CS/EC |
| 2 | Cases and controls drawn from the same population | CC |
| 3 | Participation rate >80% for cases/cohort | C/CC/CS/EC |
| 4 | Participation rate >80% for controls | CC |
| Assessment of risk factor | | |
| 5 | Exposure assessment blinded | C/CC/CS/EC |
| 6 | Exposure measured identically for cases and controls | CC |
| 7 | Exposure assessed prior to the outcome | C/CC/CS/EC |
| Assessment of outcome | | |
| 8 | BMD/Fracture assessed identically in the study population | C/CC/CS/EC |
| 9 | BMD/Presence of fracture assessed reproducibly | C/CC/CS/EC |
| 10 | BMD assessed according to absorptiometry of at least the hip and/or spine  Fracture identification assessed according to radiographic reports | C/CC/CS/EC |
| Study design | | |
| 11 | Prospective design used | C/CC/CS/EC |
| 12 | Follow-up time >24 months | C/EC |
| 13 | Withdrawals <20% | C/EC |
| 14 | Statement of ecological study design | EC |
| 15 | Justification of ecological study design | EC |
| 16 | Discussion of cross-level bias | EC |
| Analysis and data presentation | | |
| 17 | Appropriate analysis technique used | C/CC/CS/EC |
| 18 | BMD adjusted for at least age and BMI/fractures adjusted for at least age and sex | C/CC/CS/EC |

*Applicable study types: C- Cohort, CC- Case-control, CS- Cross-sectional, EC- Ecological cohort.

Table 3 Quality assessment criteria for bioarchaeological studies of bone health and socioeconomic status.

| **Item** | **Criterion** |
| --- | --- |
| 1 | Clearly stated aim |
| 2 | Primary outcome |
| 3 | Status designation and distribution |
| 4 | Condition of the sample |
| 5 | Sample preservation |
| 6 | Age-at-death distribution |
| 7 | Method for age estimation |
| 8 | Sex distribution |
| 9 | Method for sex estimation |
| 10 | Time period |
| 11 | Method of measurement |
| 12 | Exclusion criteria |
| 13 | Appropriate statistical tests and methodological standards |
| 14 | Selective or incomplete reporting |
| 15 | Comparison of data with equivalent groups |
| 16 | Historical documentation and contextual information |

*Table 4 Study characteristics, conclusions and quality assessment scores for each of the examined studies in the review of bone health and socioeconomic status.*

| Author, year | Country | Years | Measure | SES indicators and lifestyle factors | Study design | Conclusions | Quality Assessment  score |
| --- | --- | --- | --- | --- | --- | --- | --- |
| (Alver et al., 2007) | Norway | 2000-2001 | Forearm BMD | 1. Social index (accounts for unemployment, education, single parents and immigrants) in Norway – East (less affluent) to West (more affluent). 2. Education, 3. marital status, 4. BMI, physical activity, alcohol, smoking, hormone replacement therapy (HRT) 5. disability pension. | Cross-sectional | Positive association only in females | 5/10 |
| (Amin et al., 2026) | England | 2004-2025 | Lumbar spine and femoral neck BMD, major osteoporotic fractures | 1. Deprivation indices (house crowding, car, home) from postcode 2. Glucocorticoid use, previous fragility fracture, family history, smoking/alcohol, RA and BMI | Cross-sectional | Positive association | 6/10 |
| (Amiri et al., 2008) | Iran | 2004-2005 | Lumbar spine and femoral neck BMD | 1. Poverty- from financial support and minimum standard of living 2. BMI 3. Physical activity 4. Smoking   Parity, HRT | Cross-sectional | Positive association | 7/10 |
| (Borrè et al., 2015) | Italy | 8-14^th^ century | Femoral cortical index, lumbar spine and proximal femur BMD | Burial location | Archaeological | Cortical index results not significant, negative association for BMD | 13/16 |
| (Brennan et al., 2009) | Australia | 1994-1997 | Lumbar spine, total hip, forearm BMD | 1. socioeconomic indices from residential address (incl. education, occupation/income). 2. Lifestyle= calcium intake, smoking, physical activity, alcohol intake, hormone | Cross-sectional | Low BMD in lowest and highest quintiles | 6/10 |
| (Brennan et al., 2010) | Australia | 2001-2006 | Lumbar spine and femoral neck BMD | 1. residential address 2. income 3. education 4. occupation 5. Lifestyle = calcium intake, smoking, physical activity, medications, alcohol intake | Cross-sectional | Highest BMD in mid-quintile young men, lowest BMD in mid-quintile of older men | 6/10 |
| (Brennan et al., 2013) | Australia | 2002-2004 | Lumbar spine, femoral neck, total body BMD, vertebral fractures | 1. Residential areas as a collection of social indexes 2. BMI, calcium, vitamin D, smoking, alcohol, physical activity, HRT. | Cross-sectional | Positive association significant only in BMD for females, lower risk of fracture in poorer males | 6/10 |
| (Brennan et al., 2015) | Australia | 2006-2007 | Major osteoporotic fractures | 1. Residential address + census data 2. Hospital records | Cross-sectional | Positive association | 6/10 |
| (Brødholt et al., 2022) | Norway | 1050-1567 AD | Proximal femur BMD | Burial location | Archaeological | Positive association only in females | 14.5/16 |
| (Brødholt et al., 2023) | Norway | 980-1567 AD | Proximal femur BMD | Burial location | Archaeological | Negative association in young females and positive association in older females | 14/16 |
| (Crandall et al., 2012) | USA | 1995-2006 | Lumbar and spine and femoral neck BMD | 1. Childhood SE advantage (parents’ education, welfare and financial status- subjective based on memory) 2. Education 3. Subjective adult financial advantage + PIR 4. Lifestyle= smoking, alcohol, physical activity. 5. Race, age etc. | Cross-sectional | Positive association | 7/10 |
| (del Rio Barquero et al., 1992) | Spain | 1990 | Lumbar spine BMD | 1. Residential area 2. Family-head level income 3. Age, weight, height | Cross-sectional | Positive association | 6/10 |
| (Du et al., 2017) | USA | 2011-2015 | Lumbar spine and total hip BMD | 1. education 2. income 3. ethnicity 4. covariate behavioural factors | Cross-sectional | Positive association | 6/10 |
| (Elliot et al., 1996) | New Zealand | 1995 | Lumbar spine and femoral neck BMD | 1. Occupation 2. No lifestyle variables apart from calcium intake and smoking | Cross-sectional | Negative association | 5/10 |
| (Ersoy et al., 2018) | Turkey | 2018 | Lumbar spine and total body BMD | 1. SES from parents (education and income) 2. Calcium intake, Vit D intake, Physical activity | Cross-sectional | No significant associations | 7/10 |
| (Farahmand et al., 2000) | Sweden | 1993-1995 | Hip fracture | 1. marital status 2. SES from census data 3. Housing type 4. Income 5. Employment/occupation 6. Lifestyle – smoking, alcohol, physical activity, diet, BMI, hormones, parity | Case-control | Married and employed women showed less risk | 8/13 |
| (García-Quintana et al., 2024) | Spain | 2022-2023 | Lumbar spine and proximal femur BMD, major osteoporotic fractures | 1. Poverty based on annual income 2. BMI 3. Calcium intake, smoking, hrt, physical activity | Cross-sectional | Positive association | 7/10 |
| (Guilley et al., 2011) | Switzerland | 1991-2000 | Hip fractures | 1. Annual household income at area level 2. Marital status 3. No lifestyle/physiological variables. | Cohort | Positive association only in females | 7/12 |
| (Icks et al., 2009) | Germany | 1995-2004 | Hip fractures | 1. National database (population, welfare, unemployment, non-nationals, living space) | Ecological cohort | Positive association | 12/15 |
| (Johnson et al., 2019) | England | 2007-2010 | Metacarpal cortical index, distal radius fractures | 1. Ethnicity 2. Multiple deprivation index (from post-code) | Cohort | Cortical index results not significant, positive association with fracture risk | 10/12 |
| (Mahmud et al., 2024) | UK | 2006-2010 | Heel ultrasound, major osteoporotic fractures | 1. SES index from residential postcode (quantiles) 2. Age, sex, body mass, ethnicity, smoking, alcohol, supplements, physical activity | Cross-sectional | Positive association | 5/10 |
| (Miszkiewicz & Mahoney, 2016) | England | 1084-1537 AD | Microstructure, OPD | Burial location | Archaeological | Positive association | 15/16 |
| (Myong et al., 2012) | Korea | 2008-2009 | Lumbar spine and femoral neck BMD | 1. Gender 2. Menopausal status 3. BMI 4. Education level 5. Household income 6. No lifestyle variables | Cross-sectional | Positive association | 7/10 |
| (Navarro et al., 2009) | Spain | 2005-2007 | Lumbar spine and femoral neck BMD, vertebral fractures | 1. Poverty based on household income 2. BMI and diet, drug use, lifestyle and reproductive history included | Cross-sectional | Positive association for lumbar spine and fracture risk in women in poverty | 7/10 |
| (Navarro et al., 2013) | Spain | 2007-2009 | Lumbar spine BMD, vertebral fractures | 1. Poverty as defined by income 2. Calcium intake, HRT, diet, drug use (smoking, alcohol), repro history, physical activity, BMI | Cross-sectional | Positive association | 7/10 |
| (Park et al., 2016) | Korea | 2008-2012 | Vertebral fractures | 1. insurance beneficiaries vs. medical aid recipients | Cross-sectional | Positive association | 5/10 |
| (Pearson et al., 2004) | England | 2002 | Heel BMD, major osteoporotic fractures | 1. Underprivileged Area Score (unemployment, overcrowding, parents, ethnicity, mobility). 2. Smoking, alcohol, dairy products, BMI, physical activity, steroids, OP history, fracture/history, kyphosis. | Cohort | Positive association | 6/12 |
| (Petit et al., 2017) | France | 2008 | Hip fractures | 1. Deprivation Index 2. Adjusted for age and sex. | Ecological cohort | Negative association | 11/15 |
| (Quah et al., 2011) | England | 2007 | Hip fractures | 1. Multiple deprivation index (population area using post-codes) 2. No lifestyle variables | Cohort | Positive association | 11/12 |
| (Reyes et al., 2015) | Spain | 2009-2012 | Hip fractures | 1. SES composite index (unemployed/occupations, education estimated for area). 2. Smoking, alcohol, BMI. | Ecological cohort | No significant associations | 12/15 |
| (Singh et al., 2021) | India | 2017-2019 | Major osteoporotic fractures | 1. Village dwelling + conventional farming/occupation + poverty present/absent. 2. Family income, 3. BMD, calcium, vit intake | Case-control | Positive association | 8/13 |
| (Syddall et al., 2012) | England | 1939-1998 | Proximal femur BMD, major osteoporotic fractures | 1. Education level 2. Housing 3. Car availability 4. Occupation 5. Marital status 6. Smoking, alcohol, physical activity, fractures, calcium, hrt, bmi | Cohort | Positive association | 10/12 |
| (Tajik et al., 2014) | Iran | 2009 | Lumbar spine BMD | 1. Education level, income, household, occupation, 2. BMI 3. Dietary intake based on recall but suspected underreporting, reproductive factors | Cross-sectional | Positive association | 6/10 |
| (Tsai, 2019) | USA | 1999-2004 | Lumbar spine and proximal femur BMD | 1. Race/ethnicity 2. Educational level 3. Working status 4. Nativity 5. Citizenship 6. Economic status (PIR) | Cross-sectional | Positive association | 5/10 |
| (Wang & Dixon, 2006) | USA | 1988-1994 | Femoral neck and total hip BMD | 1. education 2. Poverty Income Ratio 3. behavioural factors (estrogen use, reproductive events, calcium intake, food assistance, smoking) 4. ethnicity | Cross-sectional | Positive association that was not present after health behaviours added | 7/10 |
| (Xiao et al., 2022) | USA | 2011-2020 | Lumbar spine BMD | 1. Poverty quartiles 2. Education level 3. Confounders: race/ethnicity, age, drinking, smoking, BMI | Cross-sectional | Positive association | 6/10 |
| (Yasein et al., 2016) | Jordan | 2013 | Lumbar spine and total hip BMD | 1. Marital status 2. education level 3. occupation (food choices) 4. living place 5. smoking | Cross-sectional | Positive association | 6/10 |
| (Zaki et al., 2009) | Egypt | 2687-2191 BC | Radius, lumbar spine, proximal femur BMD | Grave goods and burial location | Archaeological | Positive association in females, but negative association in males | 14/16 |
| (Zhang et al., 2023) | USA | 2011-2018 | Lumbar spine BMD | 1. Poverty income ratio 2. Education level 3. age, race, gender, smoker, blood pressure, calcium, and other blood tests. | Cross-sectional | Positive association | 6/10 |
| (Zingmond et al., 2006) | USA | 1996-2000 | Hip fractures | 1. Race/ethnicity 2. Median household income 3. acculturation | Cohort | Positive association | 6/10 |

Complete reference list of articles reviewed:

Alver, K., Søgaard, A. J., Falch, J. A., & Meyer, H. E. (2007). The Oslo Health Study: Is bone mineral density higher in affluent areas? *International Journal for Equity in Health*, *6*(1), 19. <https://doi.org/10.1186/1475-9276-6-19>

Amin, H., Khan, M. A., & Bukhari, M. (2026). Deprivation indices and their association with fragility fractures and bone density: evidence from a large observational cohort. *Rheumatology*, *65*(1), keaf550. <https://doi.org/10.1093/rheumatology/keaf550>

Amiri, M., Nabipour, I., Larijani, B., Beigi, S., Assadi, M., Amiri, Z., & Mosadeghzadeh, S. (2008). The relationship of absolute poverty and bone mineral density in postmenopausal Iranian women. *International Journal of Public Health*, *53*(6), 290–296. <https://doi.org/10.1007/s00038-008-8033-x>

Borrè, A., Boano, R., Di Stefano, M., Castiglione, A., Ciccone, G., Isaia, G. C., Panattoni, G. L., & Faletti, C. (2015). X-ray, CT and DXA study of bone loss on medieval remains from North-West Italy. *La radiologia medica*, *120*(7), 674–682. <https://doi.org/10.1007/s11547-015-0507-3>

Brennan, S. L., Henry, M. J., Wluka, A. E., Nicholson, G. C., Kotowicz, M. A., & Pasco, J. A. (2010). Socioeconomic status and bone mineral density in a population-based sample of men. *Bone*, *46*(4), 993–999. <https://doi.org/10.1016/j.bone.2009.12.029>

Brennan, S. L., Henry, M. J., Wluka, A. E., Nicholson, G. C., Kotowicz, M. A., Williams, J. W., & Pasco, J. A. (2009). BMD in Population‐Based Adult Women Is Associated With Socioeconomic Status. *Journal of Bone and Mineral Research*, *24*(5), 809–815. <https://doi.org/10.1359/jbmr.081243>

Brennan, S. L., Holloway, K. L., Williams, L. J., Kotowicz, M. A., Bucki-Smith, G., Moloney, D. J., Dobbins, A. G., Timney, E. N., & Pasco, J. A. (2015). The social gradient of fractures at any skeletal site in men and women: data from the Geelong Osteoporosis Study Fracture Grid. *Osteoporosis International*, *26*(4), 1351–1359. <https://doi.org/10.1007/s00198-014-3004-y>

Brennan, S. L., Winzenberg, T. M., Pasco, J. A., Wluka, A. E., Dobbins, A. G., & Jones, G. (2013). Social disadvantage, bone mineral density and vertebral wedge deformities in the Tasmanian Older Adult Cohort. *Osteoporosis International*, *24*(6), 1909–1916. <https://doi.org/10.1007/s00198-012-2211-7>

Brødholt, E. T., Gautvik, K. M., Benedictow, O. J., Günther, C.-C., Sjøvold, T., & Holck, P. (2023). Female skeletal health and socioeconomic status in medieval Norway (11th–16th centuries AD): Analysis of bone mineral density and stature. *International Journal of Osteoarchaeology*, *33*(1), 83–93. <https://doi.org/10.1002/oa.3178>

Brødholt, E. T., Gautvik, K. M., Günther, C.-C., Sjøvold, T., & Holck, P. (2022). Social stratification reflected in bone mineral density and stature: Spectral imaging and osteoarchaeological findings from medieval Norway. *PLOS ONE*, *17*(10), e0275448. <https://doi.org/10.1371/journal.pone.0275448>

Crandall, C. J., Merkin, S. S., Seeman, T. E., Greendale, G. A., Binkley, N., & Karlamangla, A. S. (2012). Socioeconomic status over the life-course and adult bone mineral density: The Midlife in the U.S. Study. *Bone*, *51*(1), 107–113. <https://doi.org/10.1016/j.bone.2012.04.009>

del Rio Barquero, L., Baures, M. R., Segura, J. P., Quinquer, J. S., Majem, L. S., Ruiz, P. G., Navarro, C. L., & Torné, F. M. D. (1992). Bone mineral density in two different socio-economic population groups. *Bone and Mineral*, *18*(2), 159–168. <https://doi.org/10.1016/0169-6009(92)90856-9>

Du, Y., Zhao, L. J., Xu, Q., Wu, K. H., & Deng, H. W. (2017). Socioeconomic status and bone mineral density in adults by race/ethnicity and gender: the Louisiana osteoporosis study. *Osteoporosis International*, *28*(5), 1699–1709. <https://doi.org/10.1007/s00198-017-3951-1>

Elliot, J. R., Gilchrist, N. L., & Wells, J. E. (1996). The effect of socioeconomic status on bone density in a male caucasian population. *Bone*, *18*(4), 371–373. <https://doi.org/10.1016/8756-3282(96)00006-3>

Ersoy, B., Kizilay, D. Ö., Yilmaz, S. K., Taneli, F., & Gümüşer, G. (2018). Bone mineral density, vitamin D status, and calcium intake in healthy female university students from different socioeconomic groups in Turkey. *Archives of Osteoporosis*, *13*(1), 135. <https://doi.org/10.1007/s11657-018-0482-0>

Farahmand, B. Y., Persson, P. G., Michaëlsson, K., Baron, J. A., Parker, M. G., Ljunghall, S., & for the Swedish hip fracture study, g. (2000). Socioeconomic Status, Marital Status and Hip Fracture Risk: A Population-Based Case–Control Study. *Osteoporosis International*, *11*(9), 803–808. <https://doi.org/10.1007/s001980070060>

García-Quintana, M., Tejada-Romero, M. J. G. D., Quintana-Montesdeoca, P., Hernández-Hernández, D., & Sosa-Henríquez, M. (2024). Association of poverty with higher risk of fragility fractures in postmenopausal Caucasian Spanish women. *Current Rheumatology Research*, *4*(1), 33–39. <https://doi.org/10.46439/rheumatology.4.026>

Guilley, E., Herrmann, F., Rapin, C. H., Hoffmeyer, P., Rizzoli, R., & Chevalley, T. (2011). Socioeconomic and living conditions are determinants of hip fracture incidence and age occurrence among community-dwelling elderly. *Osteoporosis International*, *22*(2), 647–653. <https://doi.org/10.1007/s00198-010-1287-1>

Icks, A., Haastert, B., Wildner, M., Becker, C., Rapp, K., Dragano, N., Meyer, G., & Rosenbauer, J. (2009). Hip fractures and area level socioeconomic conditions: a population-based study. *BMC Public Health*, *9*(1), 114. <https://doi.org/10.1186/1471-2458-9-114>

Johnson, N. A., Jeffery, J., Stirling, E., Thompson, J., & Dias, J. J. (2019). Effects of deprivation, ethnicity, gender and age on distal radius fracture incidence and surgical intervention rate. *Bone*, *121*, 1–8. <https://doi.org/10.1016/j.bone.2018.12.018>

Mahmud, M., Muscatello, D. J., Rahman, M. B., & Osborne, N. J. (2024). Association between socioeconomic deprivation and bone health status in the UK biobank cohort participants. *Osteoporosis International*, *35*(9), 1573–1584. <https://doi.org/10.1007/s00198-024-07115-3>

Miszkiewicz, J. J., & Mahoney, P. (2016). Ancient Human Bone Microstructure in Medieval England: Comparisons between Two Socio-Economic Groups. *The Anatomical Record*, *299*(1), 42–59. <https://doi.org/10.1002/ar.23285>

Myong, J.-P., Kim, H.-R., Choi, S. E., & Koo, J.-W. (2012). The Effect of Socioeconomic Position on Bone Health Among Koreans by Gender and Menopausal Status. *Calcified Tissue International*, *90*(6), 488–495. <https://doi.org/10.1007/s00223-012-9597-2>

Navarro, M. C., Sosa, M., Saavedra, P., Lainez, P., Marrero, M., Torres, M., & Medina, C. D. (2009). Poverty is a risk factor for osteoporotic fractures. *Osteoporosis International*, *20*(3), 393–398. <https://doi.org/10.1007/s00198-008-0697-9>

Navarro, M. d. C., Saavedra, P., Jódar, E., Gómez de Tejada, M. J., Mirallave, A., & Sosa, M. (2013). Osteoporosis and metabolic syndrome according to socio-economic status, contribution of PTH, vitamin D and body weight: The Canarian Osteoporosis Poverty Study (COPS). *Clinical Endocrinology*, *78*(5), 681–686. <https://doi.org/10.1111/cen.12051>

Park, S. B., Kim, J., Jeong, J. H., Lee, J.-K., Chin, D. K., Chung, C. K., Lee, S. H., & Lee, J. Y. (2016). Prevalence and Incidence of Osteoporosis and Osteoporotic Vertebral Fracture in Korea. *SPINE*, *41*(4), 328–336. <https://doi.org/10.1097/brs.0000000000001291>

Pearson, D., Taylor, R., & Masud, T. (2004). The relationship between social deprivation, osteoporosis, and falls. *Osteoporosis International*, *15*(2), 132–138. <https://doi.org/10.1007/s00198-003-1499-8>

Petit, M. P., Bryère, J., Maravic, M., Pallaro, F., & Marcelli, C. (2017). Hip fracture incidence and social deprivation: results from a French ecological study. *Osteoporosis International*, *28*(7), 2045–2051. <https://doi.org/10.1007/s00198-017-3998-z>

Quah, C., Boulton, C., & Moran, C. (2011). The influence of socioeconomic status on the incidence, outcome and mortality of fractures of the hip. *The Journal of Bone & Joint Surgery British Volume*, *93-B*(6), 801–805. <https://doi.org/10.1302/0301-620X.93B6.24936>

Reyes, C., García-Gil, M., Elorza, J. M., Fina-Avilés, F., Mendez-Boo, L., Hermosilla, E., Coma, E., Carbonell, C., Medina-Peralta, M., Ramos, R., Bolibar, B., Díez-Pérez, A., & Prieto-Alhambra, D. (2015). Socioeconomic status and its association with the risk of developing hip fractures: A region-wide ecological study. *Bone*, *73*, 127–131. <https://doi.org/10.1016/j.bone.2014.12.019>

Singh, V., Kisor Pal, A., Biswas, D., Ghosh, A., & P Singh, B. (2021). Evaluation of Environmental and Socioeconomic Factors Contri-buting to Fragility Fractures in Indians. *Journal of Environmental Science and Public Health*, *05*(03). <https://doi.org/10.26502/jesph.96120139>

Syddall, H. E., Evandrou, M., Dennison, E. M., Cooper, C., & Sayer, A. A. (2012). Social inequalities in osteoporosis and fracture among community-dwelling older men and women: findings from the Hertfordshire cohort study. *Archives of Osteoporosis*, *7*(1), 37–48. <https://doi.org/10.1007/s11657-012-0069-0>

Tajik, E., Shariff, Z. M., Esfehani, A. J., Mun, C. Y., & Ebrahimi, F. (2014). Dietary Calcium Intake and Socioeconomic Status Are Associated with Bone Mineral Density in Postmenopausal Women. *World Applied Sciences Journal*, *31*(2), 244–252. <https://doi.org/10.5829/idosi.wasj.2014.31.02.21>

Tsai, A. J. (2019). Disparities in osteoporosis by race/ethnicity, education, work status, immigrant status, and economic status in the United States. *European Journal of Internal Medicine*, *64*, 85–89. <https://doi.org/10.1016/j.ejim.2019.04.011>

Wang, M.-C., & Dixon, L. B. (2006). Socioeconomic influences on bone health in postmenopausal women: findings from NHANES III, 1988–1994. *Osteoporosis International*, *17*(1), 91–98. <https://doi.org/10.1007/s00198-005-1917-1>

Xiao, P.-L., Fuerwa, C., Hsu, C.-J., Peng, R., Cui, A.-Y., Jiang, N., Ma, Y.-G., Xu, X.-H., & Lu, H.-D. (2022). Socioeconomic status influences on bone mineral density in American men: findings from NHANES 2011–2020. *Osteoporosis International*, *33*(11), 2347–2355. <https://doi.org/10.1007/s00198-022-06498-5>

Yasein, N., Shroukh, W., Fayoumi, H., & Halaseh, L. (2016). The association between socioeconomic factors and bone mineral density in postmenopausal osteoporotic women attending family medicine clinics. *International Medical Journal (1994)*, *23*.

Zaki, M. E., Hussien, F. H., & El Banna, R. A. E.-S. (2009). Osteoporosis among ancient Egyptians. *International Journal of Osteoarchaeology*, *19*(1), 78–89. <https://doi.org/10.1002/oa.978>

Zhang, Y., Tan, C., & Tan, W. (2023). BMI, socioeconomic status, and bone mineral density in U.S. adults: Mediation analysis in the NHANES. *Frontiers in Nutrition*, *10*. <https://doi.org/10.3389/fnut.2023.1132234>

Zingmond, D. S., Soohoo, N. F., & Silverman, S. L. (2006). The role of socioeconomic status on hip fracture. *Osteoporosis International*, *17*(10), 1562–1568. <https://doi.org/10.1007/s00198-006-0161-7>
